# Investigating the psychology of eating after exercise — a scoping review

**DOI:** 10.1017/jns.2024.99

**Published:** 2025-01-27

**Authors:** Alice Porter, Russell Jago, Luke A Robles, Elin Cawley, Peter J. Rogers, Danielle Ferriday, Jeffrey M. Brunstrom

**Affiliations:** 1 NIHR Bristol Biomedical Research Centre, University Hospitals Bristol and Weston NHS Foundation Trust and University of Bristol, Bristol, UK; 2 Population Health Sciences, Bristol Medical School, University of Bristol, Bristol, UK; 3 Nuffield Department of Primary Care Health Sciences, University of Oxford, Oxford, UK; 4 Nutrition and Behaviour Unit, School of Psychological Science, University of Bristol, Bristol, UK

**Keywords:** eating behaviour, exercise, food intake, licensing, psychological compensatory eating, JBI, Joanna Briggs Institute, PRISMA, Preferred Reporting Items for Systematic Reviews and Meta-analyses, DBES, The Diet-Related Beliefs of Exercisers Scale, CEMQ, the Compensatory Eating Motives Questionnaire, CUES, the Compensatory Unhealthy Eating Scale, ESLS, the Exercise-Snacking Licensing Scale

## Abstract

Increasing food intake or eating unhealthily after exercise may undermine attempts to manage weight, thereby contributing to poor population-level health. This scoping review aimed to synthesise the evidence on the psychology of changes to eating after exercise and explore why changes to eating after exercise occur. A scoping review of peer-reviewed literature was conducted in accordance with the Joanna Briggs Institute guidance. Search terms relating to exercise, eating behaviour, and compensatory eating were used. All study designs were included. Research in children, athletes, or animals was excluded. No country or date restrictions were applied. Twenty-three studies were identified. Ten experimental studies (nine acute, one chronic) manipulated the psychological experience of exercise, one intervention study directly targeted compensatory eating, seven studies used observational methods (e.g. diet diaries, 24-h recall) to directly measure compensatory eating after exercise, and five questionnaire studies measured beliefs about eating after exercise. Outcomes varied and included energy intake (kcal/kJ), portion size, food intake, food choice, food preference, dietary lapse, and self-reported compensatory eating. We found that increased consumption of energy-dense foods occurred after exercise when exercise was perceived as less enjoyable, less autonomous, or hard work. Personal beliefs, exercise motivation, and exercise enjoyment were key psychological determinants of changes to eating after exercise. Individuals may consume additional food to refuel their energy stores after exercise (psychological compensatory eating), or consume unhealthy or energy dense foods to reward themselves after exercise, especially if exercise is experienced negatively (post-exercise licensing), however the population-level prevalence of these behaviours is unknown.

## Introduction

Four billion people globally are predicted to be living with obesity by 2035.^(1)^ Regular physical activity in combination with a healthy balanced diet is an effective strategy to prevent overweight, obesity and related non-communicable diseases, such as cardiovascular disease and type 2 diabetes,^(2)^ and is also often prescribed for weight loss.^(3,4)^ However, research shows many combined diet and exercise weight-loss interventions result in weight regain over time.^(4,5)^ Additionally, exercise training programmes often result in lower weight loss than expected, based on the prescribed exercise-induced energy expenditure.^(6,7)^ Understanding why exercise is not always as effective as expected for weight management, and developing strategies to overcome this, is important for obesity research and population-level health.

Previous research shows that a bout of exercise does not lead to a compensatory increase in energy intake,^(8)^ suggesting that acute energy depletion does not cause a compensatory (homeostatic) increase in food intake. This evidence is in line with recent theories of appetite, proposing that hunger (i.e. the desire to eat) is not driven by an immediate ‘need’ for energy (because the human body stores a large amount of energy that can be drawn from), but rather from the absence of fullness (governed largely by gut capacity), as well as environmental cues.^(9,10)^

Although humans do not need to rely on acute energy compensation after exercise to maintain long-term energy balance (i.e. a homeostatic response to an energy deficit is not required),^(9)^ in some cases, physical activity can impact energy intake and food choice, and this response may occur to a greater or lesser extent in certain individuals.^(11)^ In addition, previous research has observed variability in exercise-induced weight loss, whereby certain individuals do not lose the expected amount of weight, based on the prescribed exercise-induced energy expenditure. Several physiological and metabolic variables, such as changes to resting metabolic rate, resting heart rate, fat-free mass, and appetite sensitivity, have been proposed to explain individual variability in exercise-induced weight loss, as well as behavioural variables such as a reduction in non-exercise physical activity, non-compliance with exercise training protocols and changes in eating behaviours.^(6,12–14)^

However, less research has focused on psychological variables influencing exercise-induced energy intake. Previously, it has been shown that individuals who experience greater food reward are more likely to increase their energy intake following exercise.^(15–17)^ This work suggests that psychological factors can play an important role, which might help to explain why some people experience poorer weight-loss outcomes with exercise training.^(12,13)^

Acute experimental studies suggest that manipulating the experience of exercise to be less enjoyable or autonomous leads to an increase in subsequent energy intake, especially from energy-dense foods.^(18–20)^ In turn, this increased energy intake could undermine the negative energy balance and weight loss that can be achieved through exercise. However, studies that manipulate exercise enjoyment have not been systematically reviewed. In addition, research suggests that many people subscribe to the idea that hunger is driven by energy depletion rather than by environmental cues,^(10)^ and that they should ‘make up for’ calories lost during exercise.^(21)^ This belief, coupled with tendencies to overestimate energy expended through exercise and to underestimate energy intake,^(22,23)^ could undermine weight management goals by increasing energy intake after exercise. Research exploring whether individuals hold compensatory beliefs, how these beliefs impact changes in eating behaviours after exercise, and whether such changes are observed in free-living settings has not been reviewed systematically.

The overall aim of this scoping review was to bring together research on the psychological factors (e.g. experience of exercise, beliefs) that might affect eating after exercise. More broadly, we sought to identify trends and gaps in the literature that might inform interventions aimed at population-level weight management. Specifically, the objectives were to:

- Conceptualise and explore whether and how the psychological experience of exercise encourages changes to eating after exercise.
- Explore why eating additional food or eating unhealthily after exercise occurs, in which situations it occurs, and whether certain individuals are more susceptible to the behaviour.

## Methods

Prior to conducting this scoping review, the protocol was first published on the Open Science Framework on 19^th^ April 2023 (https://osf.io/4tsmr). We followed the Joanna Briggs Institute (JBI) guidance for conducting scoping reviews^(24)^ and the checklist for Preferred Reporting Items for Systematic Reviews and Meta-analyses (PRISMA) - extension for Scoping Reviews^(25)^ (Supplementary Table 1).

### Identification of relevant studies and search strategy

The inclusion and exclusion criteria (Table 1) were defined in terms of population, concept, context, and type of publication.^(24)^ Since literature on the effect of exercise on energy intake has been reviewed previously,^(8,26)^ we excluded experimental studies that did not include a psychological manipulation of exercise. This aligned with our objective to better understand whether psychological factors play an independent role in influencing changes to eating behaviour after a bout of exercise.

**Table 1.** Eligibility criteria

Terms	Eligibility criteria
Population	Research conducted in adults with the absence of disease and/or disorders was included. Studies in adults with overweight or obesity were included.
	Studies in athletes, children, adolescents, and animals were excluded.
Concept	Experimental research exploring eating behaviour (defined as food intake or food choice) after exercise from a psychological perspective or observational research exploring the concept of eating after exercise was included.
	Experimental studies were excluded if the psychological experience or perception of exercise was not manipulated or if imagined (rather than actual) exercise was the manipulation
	Studies were excluded if eating after exercise was combined with other health behaviours.
	Studies were excluded if they focused on sedentary behaviour or if the outcome of interest was alcohol intake or stress-induced eating.
Context	The review included research which specifically explored eating behaviour after exercise. We required the time frame of eating after exercise to be specified in the study (e.g. within 2 h of exercise).
	The review excluded studies exploring associations between dietary intake and physical activity more broadly.
Type of publication	Published empirical research studies with either quantitative or qualitative data published in English was included. No date restrictions applied.
	Reviews, protocols, opinion pieces, commentaries and conference abstracts were excluded.

To ensure the search strategy and eligibility criteria were as comprehensive as possible, a pilot search was conducted by AP and discussed with the research team. AP conducted searches of electronic databases (Medline, APA PsycInfo, Embase, Web of Science Citation Index). Search terms for the following concepts were combined with Boolean operators: after exercise AND eating behaviour AND compensatory eating. Limits were applied to exclude irrelevant studies in animals, children, athletes, and clinical populations (not including obesity). These populations were excluded due to having different dietary requirements after exercise compared to the general population. No date limit was applied. Supplementary Table 2 provides the full Medline search strategy. Searches in all databases were carried out and completed in April 2023. An updated search was carried out in September 2024 to identify any new studies which had been published since the initial search.

The titles and abstracts were extracted into the reference manager Endnote 20 for duplicate removal and then uploaded to Rayyan^(27)^ for screening. AP screened all titles and abstracts and LR/EC independently screened 20%.^(28)^ All studies that potentially met the inclusion criteria were included for full-text screening. AP conducted all full-text screening and LR/EC independently screened 25%.^(28)^ Authors were contacted when full-text articles could not be obtained. At each stage, where a clear decision about inclusion could not be made, studies were discussed with RJ and JB. No major discrepancies between reviewers were noted. To identify additional studies, the reference lists of all included articles were screened by AP, with LR independently screening reference lists for 25%.

### Data extraction and synthesis

A standardised excel spreadsheet was created for data extraction. Data extraction of three studies was piloted by AP and discussed with the research team. The extracted data included: authors, date of publication, country of study, study design, type of evidence, aims/hypotheses, recruitment and study setting, study population (sample size, age, sex, health or physical activity status, BMI or weight status, country), overview of methods, outcome variables (measure of eating behaviour), description or measure of exercise, other key variables measured, how eating after exercise was conceptualised and key study findings. Supplementary Table 3 presents the full data extraction form. AP extracted all data and LR conducted a 25% data check. Discrepancies were resolved through consensus meetings. The data on study populations, study characteristics, and key findings were charted and presented in tables and figures, and a narrative summary was provided. Following scoping review guidance, we did not appraise the methodological quality of studies.^(24)^

## Results

**Figure 1.**
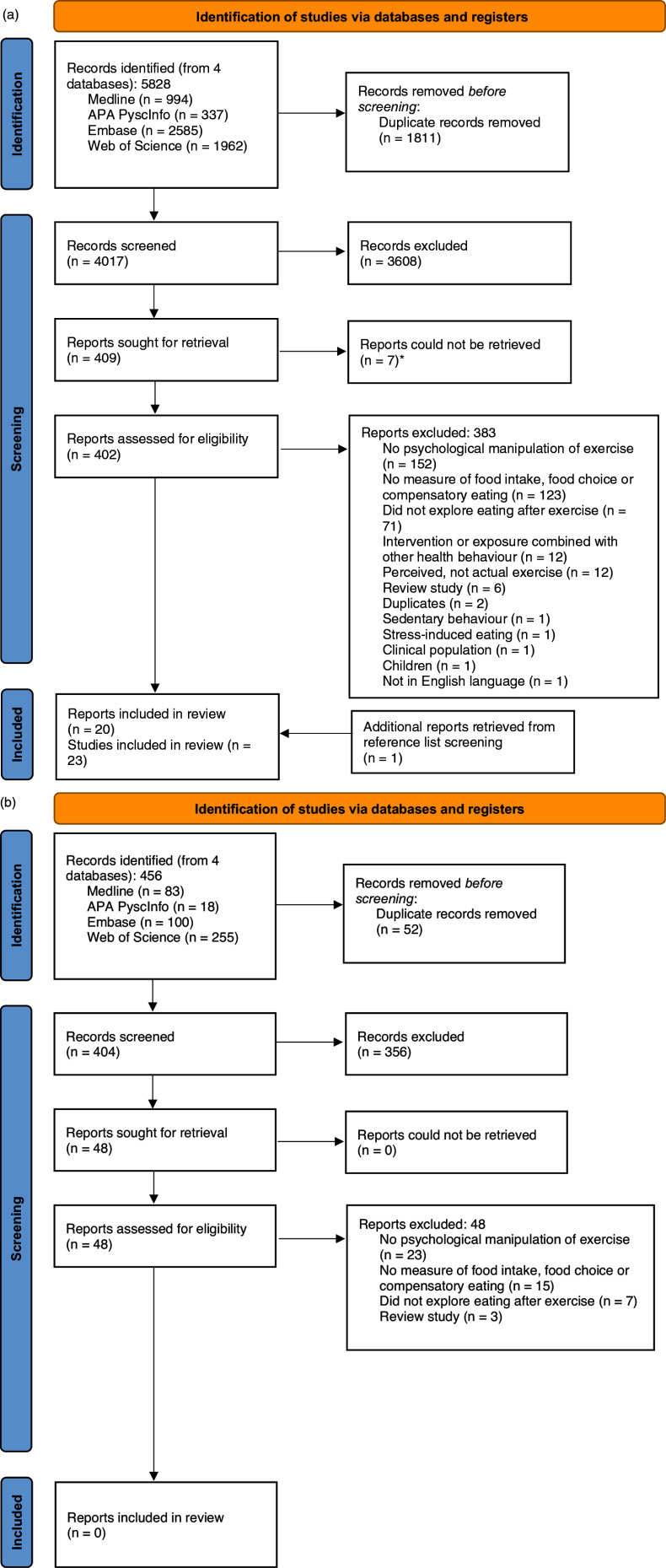
(a) PRISMA flow diagram — searches conducted April 2023. (b) PRISMA flow diagram — updated searches conducted September 2024.

Figure 1a presents the PRISMA flow diagram. After duplicate removal, 4017 articles were subject to title and abstract screening. Following exclusion, 402 were subject to full-text screening. Twenty-three studies (from 20 articles) were included in the scoping review. The main reasons for exclusion were experimental studies not including a psychological manipulation of exercise and studies not including a measure of eating behaviour. Publication dates ranged from 2014 to 2023. The updated search identified 404 additional articles (after duplicate removal), however no additional studies met the eligibility criteria for inclusion (Figure 1b).

### Descriptive summary of study populations

Table 2 presents participant characteristics across studies. Sample sizes ranged from 23 to 191 in experimental studies and 27 to 1020 in observational studies. The average age was less than 30 years in 13 studies. Most studies included both males and females (one recruited males only, three recruited females only). Of the studies reporting average BMI (n = 15), this ranged from 22.1 to 36.8 kg/m^2^ across studies. Health or physical activity status was reported in 16 studies, 10 of which described participants as healthy or active, and six described participants as inactive or having overweight/obesity.

**Table 2.** Descriptive summary of study populations

First author and date	Sample size	Average age (years)	Gender (%)	Average BMI, weight or weight status	Health or physical activity status
**Acute experimental studies**					
Alkahtani (2019)	23	20.5	100% male	BMI of 22.1 kg/m^2^	Healthy
Beer (2017)	58	22	65.5% male, 35.5% female	BMI of 22.8 kg/m^2^	Healthy
Beer (2020)	40	24.5	25% male, 75% female	BMI of 24.7 kg/m^2^	Inactive
Fenzl (2014)	96	26.1	53% male, 47% female	NR	NR
McCaig (2016)	70	28	34% male, 66% female	BMI of 23.5 kg/m^2^	Healthy, mostly lean
Panos (2018)	191	21.2	25% male, 74% female, 1% other	BMI of 23.5 kg/m^2^	65% regular exercisers
Sauchelli (2022)	34	23.5	21% male, 79% female	BMI of 22.5 kg/m^2^	Inactive
Werle (2015) (Study 1)	56	44.5	100% female	44.6% normal BMI	NR
Werle (2015) (Study 2)	46	44.4	100% female	69.9% normal BMI	NR
**Chronic experimental studies**					
Beer (2022)	36	55.9	17% male, 83% female	84.3 kg	Inactive
West (2018)	45	30.3	33% male, 67% female	BMI of 24.2 kg/m^2^	Inactive
**Observational studies of behaviour**					
Crochiere (2020)	130	52.1	15% male, 85% female	BMI of 36.8 kg/m^2^	Overweight or obese
Lyon (2014)	57	36.2	100% female	BMI of 30.2 kg/m^2^	Inactive
Maher (2020)	50	18.5	30% male, 70% female	2% underweight, 54% normal weight, 28% overweight, 16% with obesity	NR
Reily (2023) (Study 1)	48	28.7	29% male, 71% female	BMI of 24.3 kg/m^2^	Active
Reily (2023) (Study 2)	55	23.5	27% male, 73% female	BMI of 23.0 kg/m^2^	Active
Werle (2015) (Study 3)	231	35.6	65% male, 35% female	79% normal BMI	Active
West (2017)	119	20.5	53% male, 47% female	BMI of 22.2 kg/m^2^	NR
**Observational studies of motives and beliefs**					
Dohle (2015)	27	39	52% male, 48% female	NR	Active
Dohle (2021)	900	37.8	38% male, 62% female	BMI of 23.6 kg/m^2^	Active
Moshier (2016)	373	19.1	34% male, 66% female	NR	NR
Reily (2020)	804	27.5	44% male, 56% female	BMI of 25.0 kg/m^2^	Active
West (2018)	1020	30.5	35% male, 65% female	BMI of 24.9 kg/m^2^	NR

Abbreviations: NR, not reported.

### Descriptive summary of studies

Table 3 presents a summary of study characteristics and Supplementary Table 4 provides a descriptive overview. All but one study were quantitative. Of the studies that reported country of study (n = 16), most were conducted in the USA (n = 7) and Australia (n = 6). Of the studies that reported recruitment setting (n = 18), most recruited from a University (n = 9). Nine studies used an acute experimental study design to manipulate the experience of exercise (e.g. made the exercise appear more or less enjoyable, autonomous or hard work) during a single exercise bout. Two studies conducted chronic exercise training interventions with additional psychological manipulations (one included support to foster autonomy, relatedness, and competence, and one included a compensatory eating avoidance programme, involving education, goal setting, and self-monitoring). Twelve studies were observational: seven collected data on the behaviour of eating after exercise (via ecological momentary assessment (EMA), daily diaries, 24-h dietary recall, accelerometers, and pre-post exercise questionnaires) and five collected data on eating after exercise beliefs and/or motives (four via questionnaire and one via qualitative focus groups). Average exercise or physical activity duration was 15–29 min in 13% of studies, 30–60 min in 52% and not reported in 35%.

**Table 3.** Summary of study characteristics

Characteristic	N	% (of 23 studies)
**Type of evidence**		
Quantitative	22	96
Qualitative	1	4
**Country**		
Not reported	7	30
USA	7	30
Australia	6	26
UK	3	13
Saudi Arabia	1	4
Greece	1	4
Switzerland	1	4
**Recruitment setting**		
University	9	39
Not reported	5	22
Online	4	17
Multiple settings	3	13
Community setting	2	9
**Study design**		
Observational, cross-sectional	12	52
Exploring behaviour	7	58
Exploring beliefs and/or motives	5	42
Acute experimental	9	39
Chronic intervention	2	9
**Measure of exercise/physical activity** ^*^		
Acute exercise session	11	48
Exercise training programme	4	17
Device-measured (accelerometers)	3	13
Questionnaire	3	13
Daily diaries	2	9
Not reported	2	9
Observation of sport-event	1	4
**Average duration of exercise/physical activity bouts**		
15–29 min	3	13
30–60 min	12	52
Not reported	8	35
**Outcomes** ^*^		
Total energy/food intake	10	43
Energy intake from unhealthy or energy-dense foods	10	43
Energy intake from healthy foods	6	26
Self-report measure of beliefs and/or motives for post-exercise eating	5	22
Self-report measure of frequency of compensatory eating after exercise	3	13
Dietary lapse (consuming food/drink that breaks ones diet)	1	4
Portion size	1	4
Food choice	1	4
Preference for sweet and/or energy-dense foods	1	4
Dietary intake	1	4
**Measure of post-exercise eating beliefs and/or motives** ^*^		
No measure	14	61
Compensatory Eating Motives Questionnaire (CEMQ)	5	22
Exercise-Snacking Licensing Scale (ESLS)	3	13
Compensatory Unhealthy Eating Scale (CUES)	1	4
Diet-Related Beliefs of Exercisers Scale (DBES)	1	4

* Multiple measures or outcomes within some studies.

In 11 studies, participants completed an acute bout of exercise (all nine acute experimental, one chronic intervention, one observational). Acute exercise protocols were most often in the form of stationary cycling (n = 9), and ranged from 15 to 50 min, and from low to high intensity. An exercise training programme was involved in four studies (two chronic interventions, two observational). The manipulation of exercise varied across studies and included virtual reality, providing an autonomy-supportive training environment (e.g. giving participants choice and encouragement) and framing exercise in different ways (e.g. to burn 50 kcal vs 265 kcal, fat-burning vs endurance, for fun vs for exercise, for health vs for reward). Three observational studies of behaviour measured physical activity via accelerometers, two via self-reported end-of-day-diaries, and one observed a running event.

A range of outcomes were measured across studies, with several studies including more than one outcome. Total energy intake (n = 10) and intake from ‘unhealthy’ or energy-dense foods (n = 10) were the most common outcomes across studies. Food or energy intake (expressed as kcal, kJ or grams) was measured using a post-exercise laboratory test meal in 10 studies (eight acute experimental, two chronic intervention studies), with eight including a measure of energy intake from energy-dense foods. One acute study measured post-exercise dietary intake via food diaries. In the seven observational studies of behaviour, outcomes were measured using EMA (n = 2), 24-h dietary recall (n = 3), food choice between two snack items (n = 1), and a questionnaire and computerised task (n = 1). Of the five studies exploring beliefs and motives, one collected qualitative data and four developed questionnaires to explore reasons for eating after exercise. These questionnaires include the Diet-Related Beliefs of Exercisers Scale (DBES), the Compensatory Eating Motives Questionnaire (CEMQ), the Compensatory Unhealthy Eating Scale (CUES) and the Exercise-Snacking Licensing Scale (ESLS).

### Conceptualisation

The way eating after exercise was defined and described varied across studies. Table 4 presents the different definitions and how frequently these were used across studies. The terms ‘compensatory eating after exercise’ and ‘post-exercise licensing’ were used most (in 11 and 10 studies, respectively). After reviewing these definitions and study findings, we propose that the psychological influences on the tendency to change one’s eating behaviour after exercise can be conceptualised in two distinct ways: (1) ‘post-exercise psychological compensatory eating’, which captures occasions when more energy is consumed than would have been if an individual had not exercised due to holding beliefs about the need to refuel energy stores for optimal recovery and performance; and (2) ‘post-exercise licensing’, which describes consuming more energy, particularly from energy-dense foods after exercise due to the negative psychological experience of exercise or holding beliefs about the desire to reward or permit oneself to consume unhealthy food after exercise.

**Table 4.** Definitions of eating after exercise

Definitions of eating after exercise	Frequency
Compensatory eating after exercise	11
Post-exercise licensing	10
Food intake after exercise	2
Unhealthy food intake after exercise	2
Permitting spillover	1
Temporal impact of physical activity on food intake	1
Co-occurrence of physical activity and food intake	1
Post-exercise endorsement	1

### Key study findings

Table 5 presents a summary of the key findings and Supplementary Table 5 provides a descriptive overview by study. Multiple outcomes were reported in several studies, therefore, Table 5 presents the number of times an outcome was measured, and the number of times a finding was observed.

**Table 5.** Summary of key findings by study outcomes

Outcomes^*^	Number of times outcome measured	Summary of findings	Number of times finding observed
**Acute experimental studies n = 9**			
Total energy intake	6	Psychological manipulation of exercise had effect on post-exercise total energy intake	3
		Psychological manipulation had no effect on post-exercise total energy intake	3
Energy intake from unhealthy or energy-dense foods	6	Psychological manipulation of exercise had effect on post-exercise energy intake from unhealthy or energy-dense foods	4
		Psychological manipulation had no effect on post-exercise energy intake from unhealthy or energy-dense foods	2
Energy intake from healthy foods	2	Psychological manipulation had no effect on post-exercise energy intake from healthy foods	2
Dietary intake	1	Psychological manipulation had no effect on post-exercise eating behaviour	1
Preference for sweet or energy-dense foods	1	Psychological manipulation had no effect on post-exercise preference for sweet or energy-dense foods	1
**Chronic intervention studies n = 9**			
Total energy intake	2	Intervention reduced post-exercise total energy intake over time	1
		Intervention had no between-group effect on total energy intake	2
Energy intake from unhealthy or energy-dense foods	1	Intervention reduced post-exercise energy intake from unhealthy or energy-dense foods over time	1
		Intervention had no between-group effect on energy intake from unhealthy or energy-dense foods	1
Energy intake from healthy foods	1	Intervention had no between-group effect on energy intake from healthy foods over time	1
Self-reported frequency of compensatory eating after exercise	1	Intervention had no between-group effect on self-reported frequency of compensatory eating after exercise over time	1
**Observational studies of behaviour n = 7**			
Total energy intake	1	Total energy intake greater after exercise	1
Intake of unhealthy or energy-dense foods	3	Intake of unhealthy or energy-dense foods greater after exercise	1
		Intake of unhealthy or energy-dense foods lower after exercise	2
Intake of ‘healthy’ foods	3	Intake of healthy foods greater after exercise	1
		Intake of healthy foods lower after exercise	1
		No effect of exercise on intake of healthy foods	1
Dietary lapse (consuming food/drink that breaks ones diet)	1	Dietary lapse risk lower after exercise	1
Portion size	1	Portion size of meals greater after exercise	1
Food choice	1	Food choice healthier post-exercise with higher enjoyment of exercise	1
Post-exercise licensing towards unhealthy snacks	1	Post-exercise licensing towards ‘unhealthy’ snacks lower with greater autonomous motivation for exercise	1
**Observational studies of beliefs and/or motives n = 5**			
Self-reported frequency of compensatory eating after exercise	2	77% of respondents reported eating more after exercise at least sometimes	1
		36–57% of respondents reported eating more unhealthily after exercise at least sometimes	1
Beliefs about and/or motives for eating after exercise	5	Eating to reward oneself after exercise	5
		Need to eat after exercise to replenish or recover	4
		Refrain from eating after exercise	2
		Eating healthily after exercise	2
		Eating to relieve negative state	1
		Allowing oneself to eat after exercise	2
		Reduced self-control after exercise	1

* Some studies measured multiple outcomes.

#### Acute experimental studies

Five different outcomes were assessed across the nine acute experimental studies, which manipulated the psychological experience of exercise. Of the six studies that measured total energy intake, the experience of exercise was shown to have an effect in three studies. Of the six studies that measured energy intake from ‘unhealthy’ (defined by study) or energy-dense foods, the experience of exercise was shown to have an effect in four studies. Exercising whilst playing a virtual reality (VR) exergame increased exercise enjoyment and reduced post-exercise total energy intake.^(20)^ In two studies, exercise framed as ‘for fun’ rather than ‘for exercise’ led to lower consumption of an energy-dense dessert and a smaller serving of an energy-dense snack.^(19)^ Reducing participants’ autonomy whilst exercising^(18)^ and framing exercise as burning more calories (265 kcal vs 50 kcal) led to increased total and ‘unhealthy’ energy intake.^(29)^ However, the experience of exercise was not found to affect post-exercise energy intake from ‘healthy foods’ (n = 2), dietary intake (n = 1), or preference for sweet or energy-dense foods (n = 1).^(30–32)^ Fenzl et al., (2014)^(33)^ found no main effect on energy intake from an energy-dense snack when exercise was framed as ‘fat-burning’ vs ‘endurance’, however energy intake was greater in the fat-burning condition among individuals with low behavioural regulation of exercise, low positive well-being, high psychological distress, and high fatigue after exercise.

#### Chronic intervention studies

Beer et al., (2022)^(34)^ compared a 12-week sprint-interval training programme with autonomy-support (e.g. giving participants choice and encouragement) to a 12-week moderate-intensity continuous training programme without autonomy-support. West et al., (2018)^(35)^ compared an 8-week moderate to vigorous intensity training programme with and without a ‘compensatory eating avoidance programme’, which comprised an educational and goal setting workshop, and self-monitoring. Results showed a reduction in post-exercise snack intake and reduced frequency of eating after exercise, however no between-group effects in snack intake and frequency of eating after exercise were observed, respectively.

#### Observational studies of behaviour

Across the seven observational studies, two measured psychological factors and post-exercise eating behaviour. West et al., (2017)^(36)^ showed that individuals with greater autonomous motivation for exercise reported lower post-exercise licensing of energy-dense snacks. Werle et al., (2015)^(19)^ found that after a running race, individuals who reported enjoying the race were more likely to choose the ‘healthier’ snack option. The other five studies measured free-living physical activity and food intake. Lyon et al., (2014)^(37)^ found that total energy intake was greater in the 3-h after exercise compared to the same 3-h period on a non-exercise day among individuals taking part in a walking intervention. Maher et al., (2020)^(38)^ found consumption of certain ‘healthy’ (fruit, vegetables) and ‘unhealthy’ (fried fast food, sugar-sweetened beverages) foods was greater on occasions when college students took more steps than their average in the 2 h prior to eating. Crochiere et al., (2020)^(39)^ showed that the risk of dietary lapse (consuming a food or drink that breaks one’s diet) was lower when individuals participating in a weight management programme engaged in more physical activity than their average. In the two studies by Reily et al., (2023),^(40)^ active participants were less likely to consume an ‘unhealthy’ meal after exercise than on a non-exercise day, however self-reported portion size was greater after exercise.

#### Studies assessing beliefs and/or motives

Two observational studies measured the frequency of post-exercise eating via a single questionnaire item. In one, 77% of participants reported eating more after exercise at least sometimes, of whom 70% reported this occurred within 2-h of completing exercise.^(21)^ In the other study, in three separate samples, participants (36%, 51%, and 57%, respectively) reported eating ‘less healthily’ after exercise at least sometimes.^(41)^ The one qualitative study^(42)^ conducted focus groups with active individuals and identified three themes relating to eating after exercise (food as reward, nutritional replenishment to compensate for the energy and nutrients lost during exercise, and external factors leading to reduced consumption), which were used to develop and validate the Diet Related Beliefs of Exercisers Scale in another study.^(43)^ Across the four questionnaires^(21,41,43,44)^ developed to explore eating after exercise beliefs and motives, all four included a sub-scale or question relating to eating to reward oneself after exercise and three included a sub-scale or question relating to the need to eat after exercise to replenish energy or recover. Other belief and motive sub-scales were refraining from eating after exercise (n = 1), eating healthily after exercise (n = 1), eating to relieve a negative state after exercise (n = 1), allowing oneself to eat after burning energy through exercise (n = 2) and having reduced self-control after exercise (n = 1).

#### Other key measures included in studies of eating after exercise

**Figure 2.**
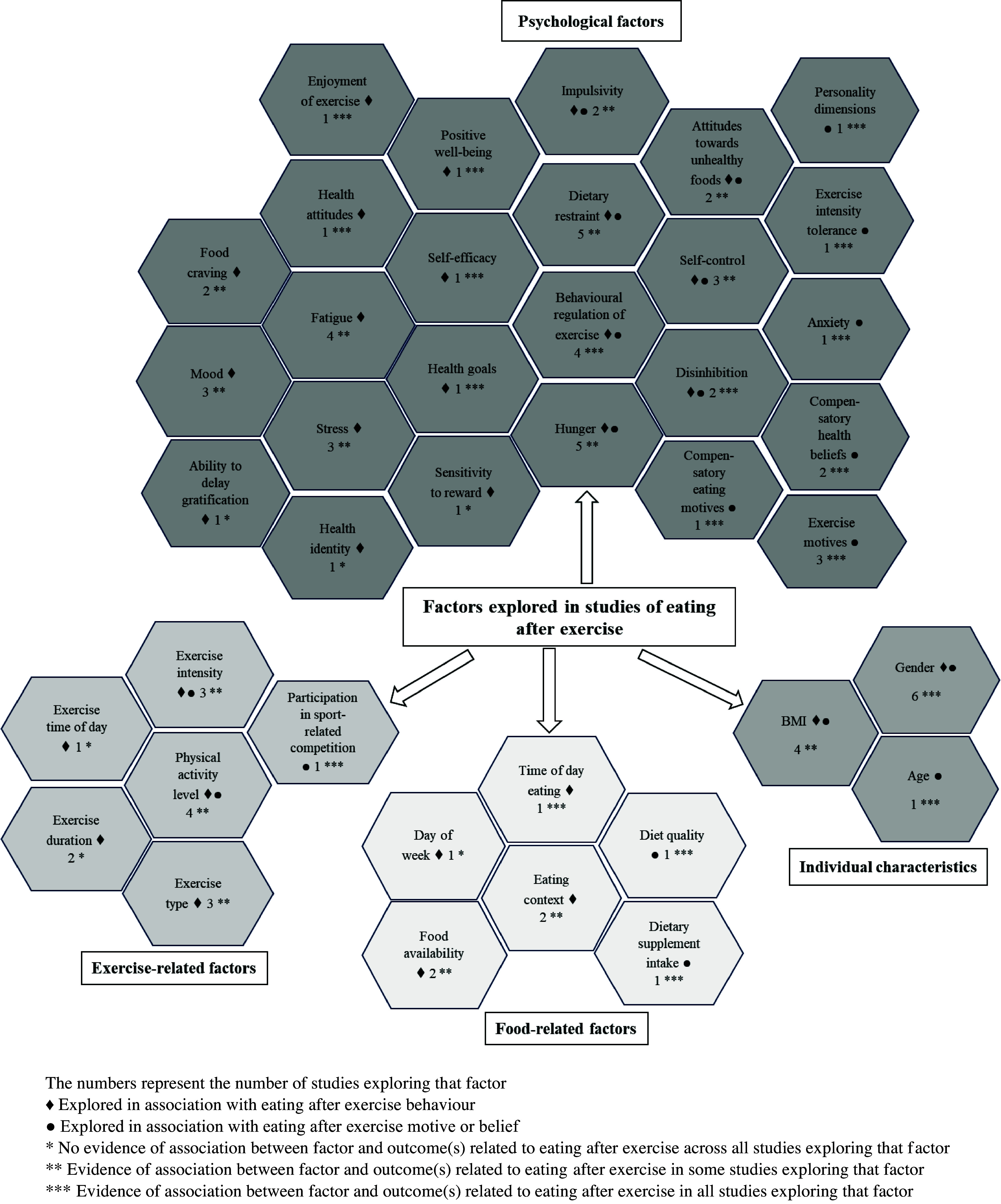
Summary of factors explored in studies of eating after exercise.

Figure 2 presents a summary of the factors that were explored in relation to post-exercise eating behaviours and beliefs across all studies. Supplementary Table 5 provides a descriptive summary by study. Twenty-five psychological factors were explored, six exercise-related factors, six food-related factors and three individual characteristics. Gender was included in six studies, all of which found gender-related differences in the post-exercise eating outcome.^(18,21,29,36,38,43)^ Findings suggest that males have greater energy intake after exercise and are more likely to eat after exercise for recovery motives. Dietary restraint was explored in five studies. Dietary restraint was positively associated with greater energy intake in an observational study^(37)^ but not in an acute experimental study.^(29)^ Dietary restraint was positively associated with post-exercise licensing in two studies^(36,41)^ but associated with the belief to eat healthily after exercise in another study.^(43)^ Behavioural regulation of exercise (a measure of exercise motivation) was associated with the post-exercise eating outcome in four studies, and exercise motives in three studies. Low behavioural regulation of exercise (i.e. low intrinsic motivation) was associated with greater ‘unhealthy’ intake and greater licensing of ‘unhealthy’ foods post-exercise.^(33,36,44)^ Greater behavioural regulation was negatively associated with beliefs about food as reward and positively associated with beliefs about healthy eating and beliefs relating to eating for replenishment post-exercise.^(43)^ Individuals who exercise for enjoyment were less likely to endorse ‘unhealthy’ snacking post-exercise and more likely to endorse consuming for recovery motives.^(21,41,44)^

## Discussion

This scoping review provides a synthesis of experimental and observational studies exploring the psychology of changes to eating after exercise. Ten of the included experimental studies (nine acute) manipulated the psychological experience of exercise, and one attempted to directly reduce compensatory eating. Seven studies aimed to observe changes to eating after exercise (e.g. eating additional or unhealthy food after exercise), and five explored beliefs towards changes to eating after exercise. Based on the literature, we have conceptualised the psychological influences of changes to eating after exercise in two ways. First, post-exercise psychological compensatory eating refers to holding beliefs about eating after exercise to refuel energy. Second, post-exercise licensing refers to eating unhealthily after exercise due to food reward beliefs or due to a negative experience of exercise. Our findings support the proposition that having a negative experience of exercise can lead to increased energy intake, especially from energy-dense foods or foods perceived to be unhealthy.

Our review showed mixed findings across studies, highlighting that there is large individual variability in whether and how individuals change their eating behaviours after exercise, and therefore how susceptible individuals are to overeating or eating unhealthily after exercise. For example, the observational evidence showed increased consumption of ‘healthy’ foods in some studies but increased consumption of ‘unhealthy’ foods in others. These mixed findings may be due to variation in measurements, populations, and study designs but may also reflect individual variability, which is often also highlighted in exercise-induced weight loss studies.^(16,17)^ Our review suggests that a key reason for this variability may due to the psychological beliefs individuals hold about eating after exercise, such as eating to refuel after exercise and eating to reward oneself, and may also be partly explained by psychological factors including exercise enjoyment and motivation.

Evidence from controlled laboratory-based exercise studies, comparing an acute exercise bout to a resting condition, shows that exercise elicits acute negative energy balance (i.e. the energy expended during the acute exercise is not fully made up for through an increase in energy intake within two hours of completing exercise).^(8)^ Some of the observational studies included in this scoping review support these lab-based findings, as evidence showed that some individuals refrain from eating or consume less energy after exercise. For example, one study found reduced consumption of ‘unhealthy’ foods on days participants exercised versus days they did not exercise,^(40)^ and one study showed increased physical activity was protective against dietary relapse when following a weight loss programme.^(39)^ Our findings support a psychological explanation for this negative energy balance, as we show that some individuals hold beliefs about refraining from eating or eating more healthily after exercise.^(42)^

In contrast, many of the acute experimental studies in this review suggest that a negative psychological experience of exercise can lead to overeating after exercise.^(18,20,29,33)^ In addition, our findings suggest that post-exercise licensing may be more common among those with low exercise enjoyment, low exercise motivation,^(19,33,36)^ those who hold beliefs about rewarding themselves with food after exercise,^(21,41,43,44)^ and when the experience or perception of exercise is manipulated in a way that leads individuals to believe they have worked harder or burnt more calories than they actually have.^(19,29,33)^ However, the two chronic experimental studies provided limited evidence that psychological support alongside exercise training can reduce overeating after exercise.^(32,35)^ It is important to highlight that the findings from the acute experimental studies suggesting individuals may overeat after exercise may not translate into chronic effects over time. As none of the included studies explored how changes to eating after exercise influence weight change over time, we cannot draw conclusions about the longer-term impacts on energy balance. A recent study by Crochiere et al. (2024)^(45)^ showed that individuals taking part in a weight loss intervention increased their energy intake before, and within two hours after physical activity; however, this was not associated with weight change. Bringing the research together, we suggest that the negative energy balance expected and potentially desired following acute exercise may sometimes be overridden by conscious actions or environmental cues. However, further research is required to explore how changing eating behaviours after exercise may impact weight management.

Our findings which suggest that exercise motivation and enjoyment are two key determinants of changes to eating after exercise aligns with two narrative reviews.^(46,47)^ Both argue that individuals with controlled motivation for exercise (e.g. exercise to obtain external reward, avoid punishment or to avoid feelings of guilt or anxiety) are more likely to engage in post-exercise licensing. This is proposed to be because individuals with controlled motivation are more likely to activate compensatory health beliefs to justify their post-exercise eating behaviour (e.g. hold the belief to reward oneself after exercise) and are less likely to exert self-control to resist tempting foods. In addition, it has been proposed that the psychological experience of exercise may affect the level of appetite hormones such as ghrelin and cortisol during exercise, however there is only very limited evidence to support this.^(46,47)^

Our findings suggesting that individual beliefs influence changes to eating after exercise, including the type of post-exercise eating (i.e. psychological compensatory eating or licensing) aligns with the wider literature on hunger beliefs. Research suggests that eating is not regulated homeostatically by detection of small meal-to-meal changes in the body’s energy resources.^(48)^ Instead ‘hunger’ (the desire to eat) can be conceptualised as having a less than full gut which, together with food liking, determines the anticipated and experienced pleasure of eating.^(9)^ However, evidence suggests that many individuals, including health professionals, believe that hunger results from an acute decrease in the body’s energy stores,^(48)^ which leads to interoceptive sensations (internal bodily events such as a rumbling stomach) that should be regarded as a cue to eat.^(10)^ The belief to consume energy after exercise to refuel energy stores that we identified in this review is directly related to this homeostatic view of hunger. For those living in obesogenic environments, these personal theories about the origin of hunger may lead to overeating,^(49,50)^ and at the same time, undermine motivation and use of exercise for weight management.^(51)^ We should note that for some individuals, such as athletes or those who exercise for goals such as to improve performance and/or strength, eating additional nutritionally balanced foods may be beneficial to aid recovery and help build muscle mass.^(52,53)^ Additionally, eating after prolonged exercise may have less impact on energy balance than shorter exercise sessions. However, for the general population using exercise to manage weight or general health, additional energy after exercise may not be required, especially if from energy dense food sources, such as high-fat high-sugar foods.

Another related but smaller body of evidence is the research on eating in anticipation of exercise. Studies by Bartucu et al.,^(54–56)^ have shown that when participants are told they will be exercising later in the day or the following day, energy intake is increased at the meal preceding exercise. However, to our knowledge, there have been no observational studies exploring whether individuals change their eating behaviours when planning exercise. In addition, further research is required to understand whether those who engage in post-exercise eating are also more likely to engage in pre-exercise eating, as the combination of both could have implications for weight management.

### Implications for future research and practice

A key research gap is the lack of knowledge about the proportion of individuals who change their eating behaviours after exercise by consuming excess or unhealthy food at a population-level. Although two of the included questionnaire studies^(21,41)^ gave some indication of prevalence by asking the questions ‘how often do you eat more after exercise?’ and ‘how often do you eat less healthily after exercise’, there is still a lack of data on how many individuals eat more, less or the same before and/or after exercise. Relatedly, Reily et al., (2023)^(40)^ found that portion sizes were larger on exercise vs non-exercise days but that healthiness did not differ, however data on how eating behaviour changes after exercise on a population level (e.g. frequency, portion size, healthiness, snacking) is still lacking. Additionally, due to the acute nature of many of the studies, less is known about how the psychological experience of exercise might impact post-exercise eating behaviours, and weight management over longer periods. Our findings highlight the need for future research exploring how eating behaviours change in response to exercise, to ascertain whether population-level interventions are required.

Our findings suggest increasing autonomous motivation for, and enjoyment of exercise may be an effective intervention for reducing licensing.^(20,46,47)^ However, in light of the barriers to engaging in exercise, such as time and cost,^(57)^ it may also be useful to develop interventions that target eating after exercise specifically, especially among those who cannot access forms of exercise they enjoy. The behaviour change techniques used in previous ecological momentary interventions and just-in-time-adaptive interventions to prevent dietary lapses^(58–60)^ and increase physical activity levels^(61)^ may provide effective methods for self-monitoring, prompting individuals and delivering feedback about eating around exercise, which could enhance the effectiveness of exercise for weight management.

Our findings highlight similarities between general hunger beliefs and beliefs specific to eating after exercise. Future research could explore whether personal hunger beliefs moderate the relationship between exercise and subsequent psychological compensatory eating or licensing. This evidence could help to inform weight management interventions, where individuals are educated about hunger and appetite after exercise, and how this may have an impact on daily energy balance.^(62,63)^ However, none of the included studies explored the impact of eating after exercise on energy balance or weight change over time, and this issue should be given priority in future research on this topic.

Our findings show that how exercise is framed can influence post-exercise licensing,^(19,29,33)^ with exercise framed as burning a large amount of calories, burning fat or labelled as being hard work leading to greater energy intake from energy-dense foods. Relatedly, research suggests smart fitness watches are not always accurate at estimating the calories expended during moderate exercise.^(64)^ In addition, smart watches often display the absolute number of calories expended during exercise rather than the additional calories expended over and above what would have been expended during rest, which may lead to individuals overconsuming after exercise. Therefore, smartwatch manufacturers may want to consider displaying this as additional information, although research into the effectiveness of this in reducing excessive energy intake after exercise is needed. In addition, the fitness industry may want to consider framing exercise in a way that promotes enjoyment and health rather than using terms such as ‘attack’ and ‘bootcamp’, although, further research in real-life fitness settings is also required.

### Strengths and limitations of the scoping review and included studies

This is the first systematic review of studies exploring the psychology of changes to eating after exercise. We followed the JBI guidelines for conducting scoping reviews^(24)^ and pre-registered our protocol (https://osf.io/4tsmr). We have drawn from studies using a range of methods, measures and outcomes to map the current literature in this field, conceptualise the psychological influences of changes to eating after exercise, and highlight gaps in the literature to inform future research and practice. However, it is important to highlight the limitations of our scoping review. We limited our search to articles published in English from academic databases, and although we did include some grey literature in the form of student theses, we did not conduct specific searches in grey literature databases. In accordance with JBI guidelines, we did not appraise the methodological quality of the included studies, which meant we could not evaluate the strength of evidence across studies. In line with our study aims, our search strategy was designed to identify studies specifically exploring the psychology of eating after exercise and observational studies exploring compensatory eating after exercise. We acknowledge, however, that there is relevant literature exploring other behavioural factors, and also physiological and metabolic variables associated with post-exercise eating, as well as experimental research not manipulating the psychological experience of exercise but instead measuring psychological factors (e.g. perceived exertion, food reward), which was not systematically captured in this review.

The majority of included studies were of acute experimental (n = 9) or observational (n = 12) design. The experimental studies provide insight into how eating behaviour may change when the psychological experience of exercise is manipulated in the short term. However, the extent to which the psychological manipulations generalise to habitual real-world experience of exercise and affect weight management over time remains unclear. We found that some observational studies reported greater consumption and some reported lower consumption after exercise. The reason for this heterogeneity in results is unclear and may be related to differences in outcome measures, including the specific way food intake was assessed, however could also be due to unmeasured moderating factors. In addition, the observational studies relied on self-report measures of food intake, which are prone to social desirability bias.^(65)^

### Conclusion

The psychological influences of changes to eating after exercise can be conceptualised in two ways: post-exercise compensatory eating and post-exercise licensing. Evidence suggests an acute bout of exercise that is perceived or experienced negatively can lead to increased consumption of energy-dense food after exercise, however, evidence on the chronic or repeated effects of exercise experience is lacking. Individuals may eat more healthily or less healthily after exercise depending on their exercise motivation, enjoyment of exercise and beliefs about eating after exercise. The two main beliefs about eating after exercise appear to be eating to refuel and eating as a reward for hard work. However, there is limited evidence on the prevalence of eating more versus eating less after exercise, on the types of foods consumed, and on the factors driving these behaviours, including how many people hold specific post-exercise eating beliefs. Clearly, there is considerable scope to do further research on eating before and after exercise to inform future interventions, and weight management practices.

## Supporting information

Porter et al. supplementary material 1 Porter et al. supplementary material

Porter et al. supplementary material 2 Porter et al. supplementary material

## Supplementary material

To view supplementary material for this article, please visit https://doi.org/10.1017/jns.2024.99
