# Supplementary material for: Investigating the psychology of eating after exercise — a scoping review
Source: J Nutr Sci. 2025 Jan 27;14:e12. doi: 10.1017/jns.2024.99 (PMC11811867; doi:10.1017/jns.2024.99)
Supplement: Porter et al. supplementary material 2 — Porter et al. supplementary material [file S2048679024000995sup002.docx]

### Supplementary Table 2. Medline Search Strategy

|  | **Concept 1a: Exercise** |
| --- | --- |
| 1. | Exercise/me, ph, px [Metabolism, Physiology, Psychology] |
| 2. | exercis*.tw,kf. |
| 3. | physical activit*.tw,kf. |
| 4. | exergaming.tw,kf. |
| 5. | (physical* adj activ*).tw,kf. |
| 6. | ((bike? or biking or bicycl* or recreational or distance) adj cycling).tw,kf. |
| 7. | (walking or running or jogging or sprinting or treadmill? or tread mill?).tw,kf. |
| 8. | physical conditioning.tw,kf. |
| 9. | (physical training or aerobic training or resistance training or strength training or cardio* training).tw,kf. |
| 10. | (exercise-induced or exercise induced).tw,kf. |
| 11. | (post-exercis* or postexercis* or post exercis*).tw,kf. |
| 12. | or/1-11 |
|  | **Concept 1b: After exercise** |
| 13. | after*.tw,kf. |
| 14. | (post or post-).tw,kf. |
| 15. | following.tw,kf. |
| 16. | subsequent.tw,kf. |
| 17. | response*.tw,kf. |
| 18. | past.tw,kf. |
| 19. | later.tw,kf. |
| 20. | or/13-19 |
| 21. | 12 and 20 |
|  | **Concept 2: Eating behaviour** |
| 22. | Eating/ph, px [Physiology, Psychology] |
| 23. | Energy Intake/ |
| 24. | energy intake.mp. |
| 25. | Feeding Behavior/ph, px [Physiology, Psychology] |
| 26. | Food Preferences/ |
| 27. | (Food adj2 choice).mp. |
| 28. | Snacks/ph, px [Physiology, Psychology] |
| 29. | (snack intake or snack consumption or snack choice).mp. |
| 30. | Diet/me, ph, px [Metabolism, Physiology, Psychology] |
| 31. | food intake.mp. |
| 32. | food consumption.mp. |
| 33. | energy balance.mp. |
| 34. | eating behavio#r.mp. |
| 35. | nutrition.mp. |
| 36. | (dietary intake or calori* intake).mp. |
| 37. | (meal size or portion size).mp. |
| 38. | (subsequent eat* or subsequent intake or subsequent food? or subsequent meal? or subsequent snack*).mp. |
| 39. | or/22-38 |
| 40. | 21 and 39 |
|  | **Concept 3: Compensatory eating** |
| 41. | compensat*.mp. |
| 42. | (compensat* adj2 eat*).mp. |
| 43. | (compensat* adj2 food).mp. |
| 44. | (compensat* adj2 behavio?r).mp. |
| 45. | (compensat* adj2 snack*).mp. |
| 46. | Compensatory Health Beliefs.mp. |
| 47. | Reward/ |
| 48. | licen*.mp. |
| 49. | (unhealth* adj3 eat*).mp. |
| 50. | (unhealth* adj3 intake).mp. |
| 51. | (unhealth* adj3 consum*).mp. |
| 52. | (unhealth* adj3 food?).mp. |
| 53. | (unhealth* adj3 snack*).mp. |
| 54. | (unhealth* adj3 diet*).mp. |
| 55. | (eat* adj3 motivat*).mp. |
| 56. | (motivat* adj3 food).mp. |
| 57. | (overeat* or over-eat or over eat).mp. |
| 58. | (overconsum* or over consum* or over-consum*).mp. |
| 59. | (excess* adj2 eat*).mp. |
| 60. | (excess* adj2 food).mp. |
| 61. | (excess* adj2 intake).mp. |
| 62. | (excess* adj2 consum*).mp. |
| 63. | (excess* adj2 snack*).mp. |
| 64. | (high fat or high-fat or high sugar or high-sugar).mp. |
| 65. | (energy dense or energy-dense).mp. |
| 66. | indulge*.mp. |
| 67. | (diet* adj2 lapse*).mp. |
| 68. | or/41-67 |
|  | **Limits** |
| 69. | Child/ not Adults/ |
| 70. | (child* or boys or girls or kids or juvenil* or minors or paediatric* or pediatric* or schoolchild*).ti. |
| 71. | (infant* or adolesc* or teen* or young adult* or toddler? or school).ti. |
| 72. | (pre-school* or preschool*).ti. |
| 73. | exp Animals/ not Humans/ |
| 74. | ((animal model* or mouse or mice or murine* or rat or rats or rodent* or muridae or murids or rabbit* or leporine* or leporidae or guineapig* or cavies or caviidae or hamster* or cricetidae or gerbil* or gerbillinae or cat or cats or feline* or felidae or dog or dogs or canine* or canidae or pig or pigs or piglet* or minipig* or swine* or porcine* or suidae or horse or horses or donkey or donkies or burros or asses or equine* or equidae or sheep or lamb or lambs or ovine or ovidae or goat or goats or cow or cows or cattle or bovine* or bovidae or primate* or monkey or monkeys or macaque or macaques or marmoset or marmosets or bat or bats) not human*).ti. |
| 75. | (athlete? or elite or sportsm?n or sportswom?n).ti. |
| 76. | (cancer or neurological disease or Alzheimer's or dementia or Parkinson's or Schizophrenia).ti. |
| 77. | pregnan*.ti. |
| 78. | osteoporo*.ti. |
| 79. | clinical.ti. |
| 80. | (eating disorder? or disordered eating or bulimia or bulimic or anorex* or binge-eating or binge eating or purge or purging).ti. |
| 81. | or/69-80 |
|  | **Results** |
| 82. | 40 and 68 |
| 83. | 82 not 81 |
